# Developmental plasticity under human management shaped cereal evolution prior to domestication in the Early Holocene southern Levant

**DOI:** 10.1073/pnas.2535274123

**Published:** 2026-08-04

**Authors:** Jade Whitlam, Pascal Flohr, Amy Bogaard, Michael Charles, Bill Finlayson, Cheryl A. Makarewicz

**Affiliations:** ^a^ School of Archaeology, University of Oxford, Oxford OX1 3TG, United Kingdom; ^b^ Department for Continuing Education, University of Oxford, Oxford OX1 2JA, United Kingdom; ^c^Institute for Prehistoric and Protohistoric Archaeology, University of Kiel, Kiel D-24118, Germany; ^d^Sante Fe Institute, Sante Fe, NM 87501; ^e^Archaeology Stable Isotope Laboratory, University of Kiel, Kiel D-24118, Germany

**Keywords:** southwest Asia, Neolithic, domestication, origins of agriculture, stable carbon isotope analysis

## Abstract

The domestication of plants in southwest Asia was an evolutionary process that took place over several millennia in the Early Holocene. During this time, domestic species developed distinct traits that distinguished them from their wild counterparts. Current models of plant domestication emphasize the role of genetic selection in the evolution of these traits, viewing these as heritable adaptations that arose in response to selective pressures associated with human cultivation. In cereals, domestication resulted in the evolution of nonshattering rachis and increased grain size, two traits that can be tracked directly in the archaeobotanical record. Analyses of Early Neolithic cereal remains demonstrate that grain size increase occurred prior to the evolution of nonshattering rachis, a sequence that is often interpreted as evidence of selection for larger grains under cultivation, specifically tillage. Here, we combine morphological and metrical analyses of cereal remains, stable carbon isotope analysis, and weed ecology to test this hypothesis, using three assemblages from the southern Levant: Pre-Pottery Neolithic A Sharara (*c.* 9250–9200 cal BCE), Pre-Pottery Neolithic A el-Hemmeh (*c.* 9400–8700 cal BCE), and Late Pre-Pottery Neolithic B el-Hemmeh (*c.* 7500–7000 cal BCE). Our findings indicate that increased grain size in the Early Holocene can be better understood as a plastic response to variation in growing conditions, specifically moisture, rather than as a result of genetic selection for increased grain size under cultivation. We propose that cereal evolution in southwest Asia was initially driven by developmental plasticity, followed by genetic selection.

## Significance

Cereals were among the earliest crops domesticated in southwest Asia and remain central to global agriculture. We can track cereal domestication archaeologically using a suite of morphological traits that distinguish domestic species from their wild counterparts. The process by which these traits evolved is still not fully understood but has broadly been framed as genetic adaptation to cultivation. Here, we seek to disentangle how two key domestication traits relating to grain dispersal and grain size evolved. By taking a multistranded approach to recent archaeobotanical evidence, we demonstrate that in the Early Holocene southern Levant, aspects of cereal domestication unfolded in a sequence where phenotypic change linked to varying water availability, rather than to tillage, preceded genetic change associated with cultivation.

Plant domestication is an evolutionary process in which wild species adapt to human-managed environments and the novel selection pressures they impose. This has resulted in domestic crops that exhibit distinct traits compared to their wild counterparts. While the adaptive traits selected for during domestication vary across species, convergent evolution has led to multiple crop species acquiring similar traits, known collectively as the “domestication syndrome” (1). For cereal crops, domestication syndrome traits include loss of shattering (indehiscence), increased grain size, loss of dispersal aids (e.g., awns, hairs), reduced seed dormancy and apical dominance (1–3). Loss of shattering and increased grain size are key traits for domestication studies, as they can be directly identified in the archaeobotanical record (4).

A widely held prerequisite for the domestication of crops was the cultivation of their wild progenitors, termed “predomestication cultivation” (PDC). PDC created the selective environments in which domestic traits evolved and has been inferred at more than a dozen sites across the Fertile Crescent, based on multiple lines of evidence, including increased seed size and the occurrence of arable weeds presumed to reflect tillage (5–14). However, recent ecological analysis of early weed flora has shown that systematic annual tillage did not characterize PDC (15), suggesting that these early management systems were distinct from later farming and require redefinition. More broadly, a pervasive focus on genetic selection as central to domestication has underpinned interpretation of seed size increases, with little attention paid to developmental plasticity [i.e., the ability of a genotype to produce different phenotypes in response to environmental conditions (16)].

Here, we present a multistranded approach, integrating morphological and metrical analysis of cereal remains with stable carbon isotope analysis and functional weed ecology to investigate the nature of PDC and to evaluate the role of developmental plasticity and genetic selection in directing cereal evolution. Our results shed light on the ecological conditions, human practices, and evolutionary processes that shaped cereal domestication in the southern Levant, where long-term archaeological investigation has framed wider understanding of the agricultural transition.

## Cereal Domestication in Southwest Asia.

The principal cereal crops domesticated in southwest Asia during the Early Holocene (*c.* 9700–6000 cal BCE), include barley (*Hordeum vulgare*), and the glume wheats emmer (*Triticum turgidum* subsp. *dicoccum*) and einkorn (*Triticum monococcum*). It is widely accepted, based on archaeobotanical and genomic data, that plant domestication was a multicentric and protracted process, involving rates of evolution comparable to those observed under natural selection (17, 18). Archaeobotanical data also show that the full suite of domestication syndrome traits did not emerge simultaneously in cereals but were subject to changing rates of evolution over time and between different regions (4, 19, 20). Increased grain size appears to have preceded the development of indehiscence, with larger grains thought to reflect cultivation and domestication reported from numerous Pre-Pottery Neolithic A (PPNA: 9700–8700 cal BCE) sites (4, 21, 22). In contrast, it is not until the Late Pre-Pottery Neolithic B (LPPNB)-Early Pottery Neolithic (7500–6300 cal BCE) that nonshattering (indehiscent) cereal ears become dominant at sites across the Fertile Crescent, signaling the fixation of this trait in cereal populations (23, 24).

In wheat and barley, loss of shattering is known to have involved mutations in the *BTR1* and *BTR2* genes (20, 25). Selection for nonshattering mutants has been linked to practices such as delayed harvesting, annual resowing of grain from household stores, and later, systematic sickle harvesting (26, 27), all of which would have conferred an evolutionary advantage on plants that retained their seeds when ripe. In contrast, the genetic mechanisms underlying the evolution of increased grain size are more complex, and the practices that selected for this trait remain poorly understood. Tillage has been hypothesized to have played a role in selection for increased grain size, by creating deeper burial conditions in which larger seeds have a competitive advantage (2, 4, 28). However, this hypothesis is not supported by experimental data (29, 30) and has yet to be systematically evaluated archaeologically.

Studies of the evolution of grain size are further complicated by the plastic nature of this trait (31, 32). Developmental plasticity refers to the ability of a single genotype to alter its developmental processes and phenotypic outcomes in response to different environmental conditions, often in ways that are functionally adaptive and which enhance plant fitness within that environment (33–35). In recent years, interest in developmental plasticity as a novel evolutionary process has increased, with proponents of the Extended Evolutionary Synthesis (EES) regarding plasticity as one of several processes that shape evolution by generating and directing variation on which genetic selection operates (36–39).

In the context of plant domestication, it has been proposed that plasticity enabled plants to adjust to the cultivated environment rapidly, and to express phenotypes that were advantageous to humans, before corresponding genetic selection took place (37, 39–42). In this way, plasticity may have directed crop progenitors toward domestic phenotypes, encouraging more intensive human engagement with these species and promoting the selection of genotypes that acted to fix these traits (37, 40, 43). Proponents of the EES further contend that persistent plastic traits can be transformed to become fixed components of an organism’s genome through genetic assimilation, a process in which environmentally induced traits become genetically encoded in stable environments due to the high costs of maintaining plasticity (39). In this way phenotypic change in domestication syndrome traits may have preceded and even facilitated genetic change.

Here, we report on results from two Early Holocene sites in the southern Levant, where well-preserved archaeobotanical evidence supports the hypothesis that cereal evolution was shaped by developmental plasticity, as well as genetic selection, raising the possibility that phenotypic outcomes may have influenced subsequent genetic changes. Our findings also provide direct archaeological evidence that increased grain size did not develop as a genetic adaptation to deeper burial associated with tillage, but instead reflects a plastic response to the relatively wet conditions under which some harvested cereal stands grew. This suggests that PDC, rather than resembling later forms of cultivation, comprised a distinct suite of practices, offering a new basis for understanding an elusive episode in the domestication story.

## Study Sites.

**Fig. 1.**
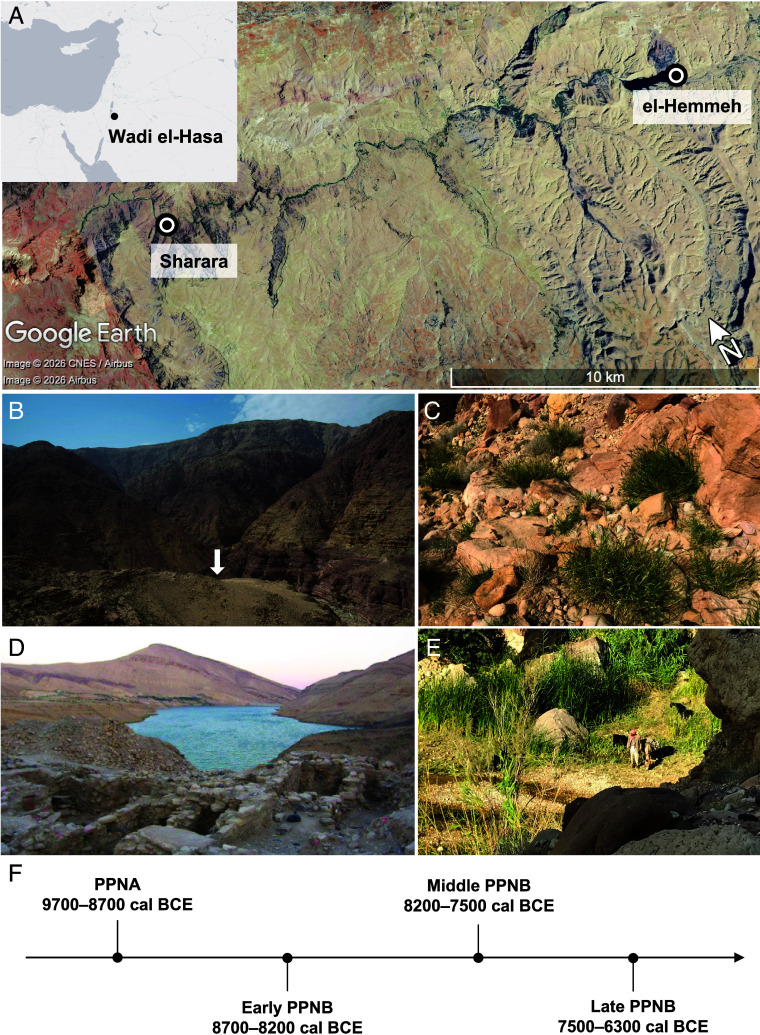
Setting of archaeological sites. (*A*) Google Earth map showing the location of Sharara and el-Hemmeh in the Wadi el-Hasa, with map showing location of Wadi el-Hasa in southern Levant inset. (*B*) Position of Sharara, indicated by the arrow, on a high outcrop in the Wadi el-Hasa. (*C*) Sparse vegetation growing on rocky slopes around Sharara. (*D*) Position of el-Hemmeh on an alluvial terrace overlooking the wadi, now flooded by modern damming. (*E*). Rich vegetation growing around well-watered wadi edges at Sharara. (*F*) Timeline for Pre-Pottery Neolithic (PPN).

The southern Levant is an important region for the study of early cereal management and domestication in southwest Asia. Archaeobotanical evidence indicates that wild barley (*Hordeum spontaneum*) and wild emmer wheat (*Triticum dicoccoides*) were brought into cultivation and independently domesticated here in the Early Holocene (23). The archaeological sites of Sharara and el-Hemmeh, located 25 km apart in the Wadi el-Hasa in southern Jordan (Fig. 1), provide a valuable opportunity to examine how barley and emmer were managed at a local level during the Early Holocene and to track changes in their domestication status.

The PPNA settlement of Sharara (*c.* 9250–9200 cal BCE) is situated on a rocky slope, high above the narrow wadi floodplain (−100 m a.s.l.) and consists of a loose scatter of stone structures spread over an area of *c.* 0.5 ha. El-Hemmeh, located upstream of Sharara and situated on an alluvial terrace fragment overlooking a wider section of the wadi floodplain (450m a.s.l.), was inhabited during the latter half of the PPNA (c. 9400–8700 cal BCE) and in the LPPNB (*c.* 7500–7000 cal BCE). The PPNA settlement supported a relatively dense configuration of free-standing and semisubterranean circular, and semicircular, stone and mud structures (44, 45), while the LPPNB settlement consisted of agglutinative rectilinear stone architecture reaching several meters in height (45, 46).

The PPNA inhabitants of both settlements exploited a range of wild cereals, focusing primarily on barley, along with emmer wheat, pulses, figs (*Ficus carica*), and pistachio (*Pistacia* sp.) (13, 47, 48). In contrast, emmer wheat was the primary cereal crop at LPPNB el-Hemmeh, while barley was exploited at low levels along with einkorn wheat (*SI Appendix*, Fig. S1). PDC of barley has previously been inferred at PPNA el-Hemmeh based on the large size of cereal grains, the presence of purported arable weeds, and the identification of rachis remains with “ripped scars,” which were suggested to result from the harvesting and processing of barley while it was still green (13).

In this study, we report on additional analyses undertaken at both sites, including i) morphological analysis of barley and emmer rachis remains to track the loss of shattering; ii) metrical analysis of barley grains to document increases in grain size; iii) single-grain stable carbon isotope analysis of barley and glume wheat (emmer and einkorn) to infer the moisture conditions under which cereals were grown; and iv) weed ecological analysis to reconstruct the conditions of cereal cultivation in relation to tillage.

## Morphological Analysis of Cereal Rachis.

**Fig. 2.**
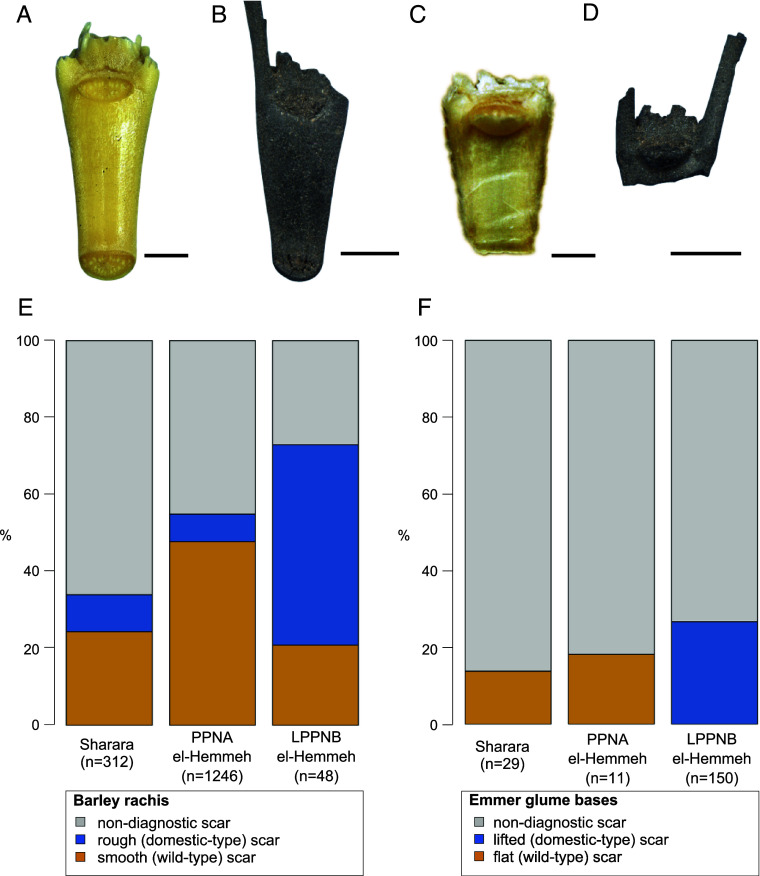
Rachis morphotypes, all scale bars 1 mm. (*A*) Modern example of wild barley rachis internode showing smooth abscission scar. (*B*) Archaeological specimen from el-Hemmeh with smooth (wild-type) abscission scar. (*C*) Modern example of domestic barley rachis showing rough abscission scar. (*D*) Archaeological specimen from el-Hemmeh with rough (domestic-type) abscission scar. (*E*) Percentages of different barley rachis morphotypes at PPNA Sharara, PPNA el-Hemmeh and LPPNB el-Hemmeh, with total numbers of specimens shown. (*F*) Percentages of different emmer rachis morphotypes (quantified as glume bases that retain the abscission scar) at PPNA Sharara, PPNA el-Hemmeh, and LPPNB el-Hemmeh with total numbers of specimens shown.

Wild wheat and barley ears spontaneously shatter upon maturity, producing rachis morphotypes characterized by smooth abscission scars (22, 49) (Fig. 2*A*). In contrast, domestic wheat and barley ears, which have evolved to remain intact upon ripening and require mechanical action (i.e., threshing) to release their grain, produce rachis morphotypes characterized by rough abscission scars (Fig. 2*C*). The relative proportions of smooth and rough scars within a cereal assemblage therefore provide a means of assessing domestication status. For emmer wheat, domestic status can be securely identified by examining the longitudinal section of the upper rachis abscission scar: in shattering (wild) emmer this appears flat in orientation, whereas in nonshattering (domestic) emmer the scar appears lifted from the internode (50).

For rachis remains recovered from Sharara and el-Hemmeh, barley abscission scars were classified as smooth, rough, or nondiagnostic, and for emmer wheat, as flat, lifted, or nondiagnostic (*Materials and Methods* and *SI Appendix*, *Supporting Information Text*). Fig. 2 illustrates the relative proportions of the different rachis morphotypes for barley and emmer within each assemblage.

These results clearly demonstrate that both barley and emmer were morphologically wild at PPNA Sharara and PPNA el-Hemmeh, with no lifted scars identified among emmer remains and only low proportions of rough scars observed in barley. The presence of a small proportion of nonshattering rachis in wild barley populations is consistent with previous studies reporting the minor occurrence of rough scars (*c.* 10%) in modern wild barley (51). In contrast, at LPPNB el-Hemmeh, both barley and emmer wheat appear morphologically domesticated, based on the high proportions of rough and lifted scar morphotypes present. This indicates that nonshattering populations of both species were being maintained under cultivation at this time, consistent with evidence from other Middle/LPPNB sites in the Levant (23).

## Metrical Analysis of Cereal Grain.

**Fig. 3.**
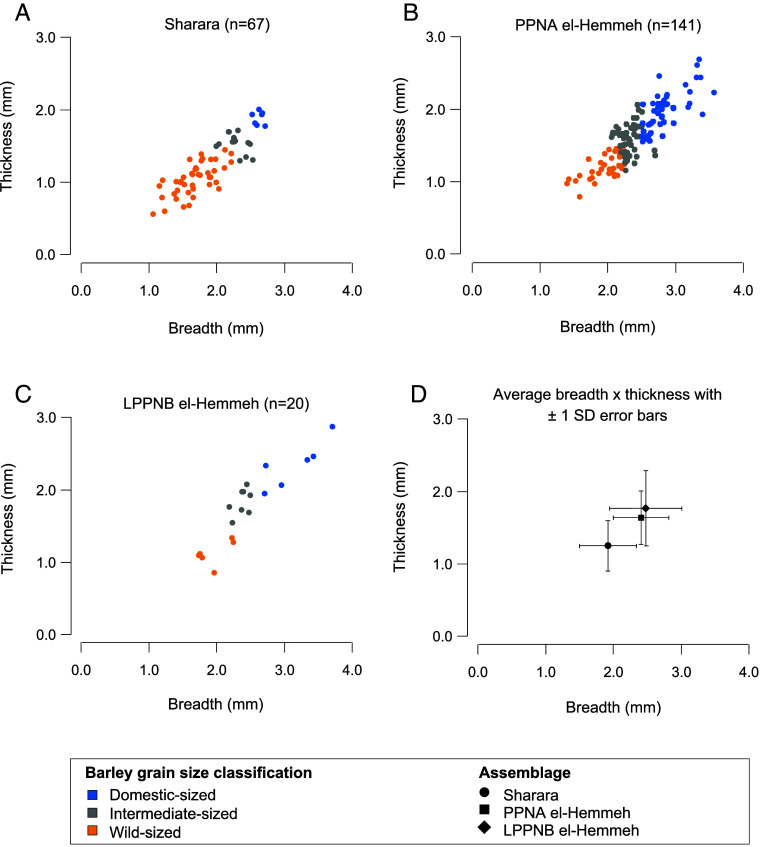
Scatterplots showing the breadth and thickness of 228 individual barley grains coded according to whether grains were classified as “wild-sized,” “intermediate-sized,” or “domestic-sized.” (*A*) PPNA Sharara. (*B*) PPNA el-Hemmeh. (*C*) LPPNB el-Hemmeh. (*D*) Average grain breadth and thickness (±1 SD) for PPNA Sharara, PPNA el-Hemmeh, and LPPNB el-Hemmeh populations. Error bars represent within-population variation in each dimension.

The occurrence of large-sized cereal grains at PPN sites has been interpreted as evidence for domestication, with thresholds of grain breadth and grain width used to distinguish wild grains from cultivated/domestic grains (e.g., refs. 47, 49). To explore variation in barley grain size at our sites, we measured the breadth and thickness of all grains and grain fragments. Grains were subsequently classified into three descriptive categories, “wild-sized,” “intermediate-sized,” and “domestic-sized,” based on established biometrical parameters (*Materials and Methods* and *SI Appendix*, *Supporting Information Text*). The results are presented in Fig. 3. The low number of emmer wheat grains from PPNA Sharara and PPNA el-Hemmeh precluded metrical analysis of this taxon.

Our results demonstrate that barley grains vary widely in size, both within and between sites, with all three assemblages producing grains classified as “wild-sized,” “intermediate-sized,” and “domestic-sized” (Fig. 3). The smallest grains were identified at Sharara, where the average grain size (1.91 ± 0.42 mm × 1.25 ± 0.35 mm; Fig. 3*D*) falls firmly within the “wild-sized” range. In contrast, both el-Hemmeh assemblages produced larger grains, with mean grain sizes of 2.40 ± 0.41 mm × 1.64 ± 0.37 mm for the PPNA, and 2.47 ± 0.53 mm × 1.77 ± 0.52 mm for the LPPNB (Fig. 3*D*), both falling within the “intermediate-sized” range. While LPPNB el-Hemmeh grains were slightly larger on average than their PPNA counterparts, it is notable that both el-Hemmeh assemblages yielded a similar proportion of grains within the “wild-sized” and “domestic-sized” categories, despite rachis morphologies indicating a shattering (wild) barley population at PPNA el-Hemmeh and a nonshattering (domestic) population at LPPNB el-Hemmeh. This suggests that grain size does not correlate directly with domestication status and that other factors, such as population-level variation and plastic responses to growing conditions, are driving the observed pattern.

## Stable Carbon Isotope Analyses.

To assess whether growing conditions influenced grain size variation, we undertook stable carbon isotope analysis of barley grains from Sharara and el-Hemmeh. We also analyzed glume wheat (emmer and einkorn) grains from el-Hemmeh to compare variation among cereal species. Sharara did not produce enough well-preserved glume wheat grains to permit isotopic analysis. Stable carbon isotope analysis provides a means of inferring the different moisture conditions under which grains developed, with higher stable carbon isotope discrimination (Δ^13^C) indicating greater water availability and lower Δ^13^C values reflecting lower availability and hence water stress (52, 53). Water stress during grain development is one of the few situations where plasticity relating to grain size is the principal determinant of yield variation in cereals, rather than grain number (54). Stable carbon isotope discrimination thus provides a reliable proxy for differences in water availability that are likely to have directly affected grain size.

**Fig. 4.**
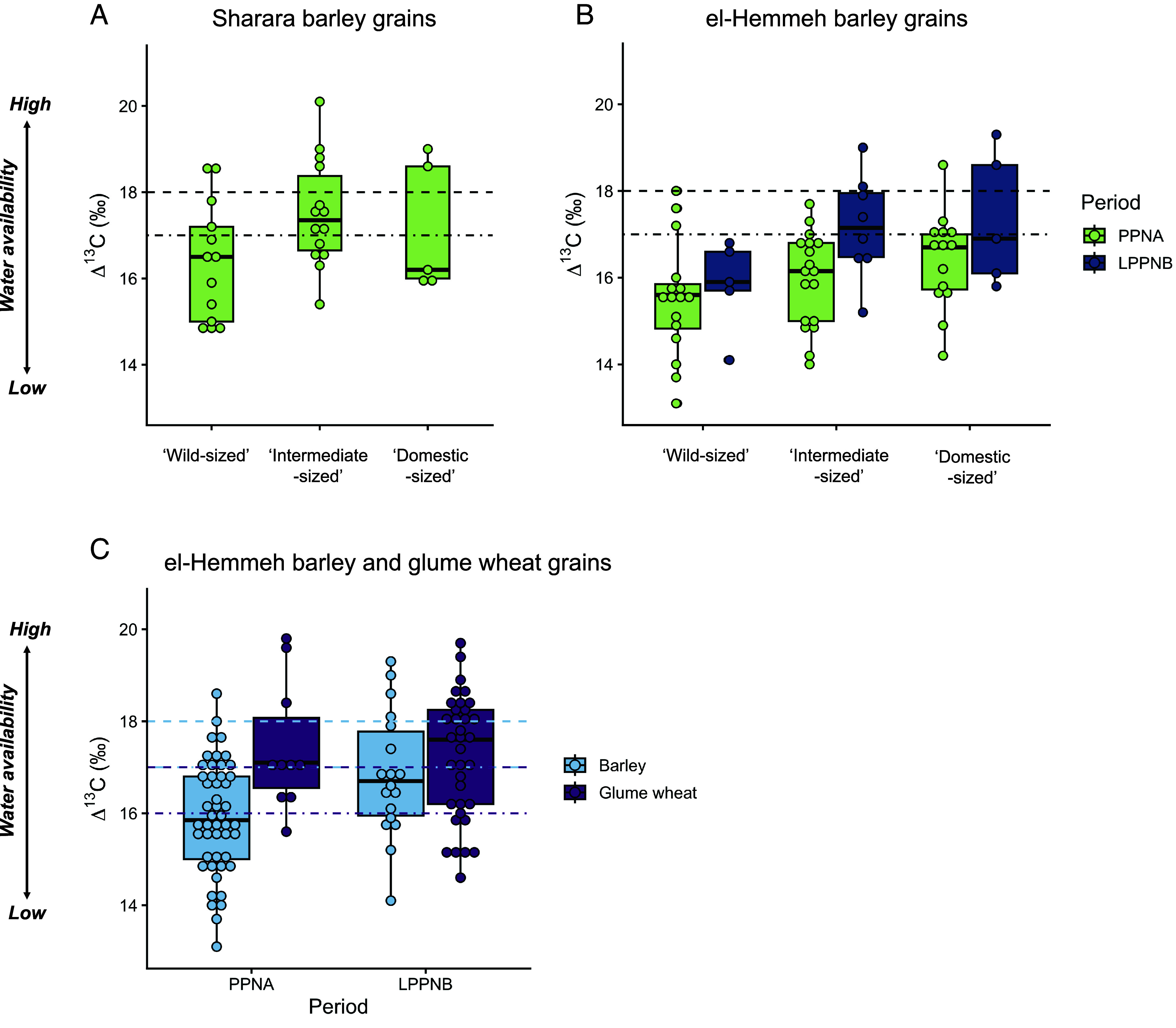
Box plots of carbon stable isotope discrimination with circles for individual grain values, showing Δ^13^C in ‰ by size classification as described in text. (*A*) PPNA barley grains from Sharara. (*B*) PPNA (light green/light) and LPPNB (blue/dark) barley grains from el-Hemmeh. In both A and B, the dashed lines indicate the approximate boundary for well-watered plants based on modern values (above 18‰) and water-stressed plants (below 17‰) [(53), see also ref. 52]. (*C*) el-Hemmeh barley (light blue/light) and glume wheat (purple/dark) for both the PPNA (*Left*) and the LPPNB (*Right*). The horizontal lines indicate the approximate well-watered/poorly watered boundaries for barley (light blue) and wheat (purple, well-watered above 17‰ and water-stressed below 16‰).

Single-grain Δ^13^C values are shown in Fig. 4. For barley these are grouped according to the previously defined “wild-sized,” “intermediate-sized,” and “domestic-sized” categories (Fig. 4 *A* and *B*; see *SI Appendix*, *Supporting Information Text*). For el-Hemmeh, single-grain Δ^13^C values are also grouped into barley and glume wheat categories according to period (Fig. 4*C*).

Our results show that “domestic-sized” barley grains generally have higher Δ^13^C values than “wild-sized” grains, indicating that they developed under comparatively moist conditions, though there is considerable overlap (Fig. 4*B*). At el-Hemmeh, a significant but weak correlation between Δ^13^C and barley grain size (*P* < 0.001; r^2^ = 0.24) supports the inference that water availability influenced grain size but was not the sole contributing factor. A similar, nonsignificant trend occurs at Sharara, where smaller “wild-sized” grains show lower (drier) Δ^13^C values than “domestic-sized” grains (Fig. 4*A*).

Our results also demonstrate that average Δ^13^C values for barley at Sharara are approximately 1‰ higher than those recorded for PPNA el-Hemmeh (17.0‰ ±1.4 at Sharara compared to 15.9‰ ±1.2 at el-Hemmeh; significant at *P* = 0.0009 in a two-sided *t* test, see *SI Appendix*, Tables S8 and S10), indicating that barley grew under wetter conditions here despite the smaller average grain size. This suggests that while plastic responses to water availability influenced grain size *within* a population, developmental plasticity alone cannot explain the variation in grain size observed *between* populations.

Compared to PPNA el-Hemmeh, the barley from LPPNB el-Hemmeh has significantly higher Δ^13^C values (two-sided *t* test *P* = 0.018, PPNA 15.9‰ ±1.2, LPPNB 16.8‰ ±1.4), suggesting improved moisture availability in the later period. As climatic and environmental conditions appear to have been stable and relatively wet throughout the Early Holocene (55, 56), this may reflect an improvement in growing conditions relating to cultivation, consistent with rachis evidence for the establishment of domestic cereal populations as discussed above.

Glume wheats at PPNA el-Hemmeh also have significantly higher Δ^13^C values than barley (*t* test, *P* = 0.02, wheat 17.4‰ ±1.4, barley 15.9‰ ±1.2), while at LPPNB el-Hemmeh glume wheats and barley have similar Δ^13^C values (wheat 17.2‰ ±1.3, barley 16.8‰ ±1.4) (Fig. 4*C* and *SI Appendix*, Table S8). This suggests that glume wheats grew under different conditions than barley at both PPNA and LPPNB el-Hemmeh, since in modern studies barley tends to exhibit higher Δ^13^C values when growing under the same conditions as glume wheats, reflecting its greater drought tolerance (52, 53, 57). However, based on stable carbon isotope analysis we cannot conclude whether this was due to deliberate planting or harvesting from different areas or in different years.

## Weed Ecological Analyses.

**Fig. 5.**
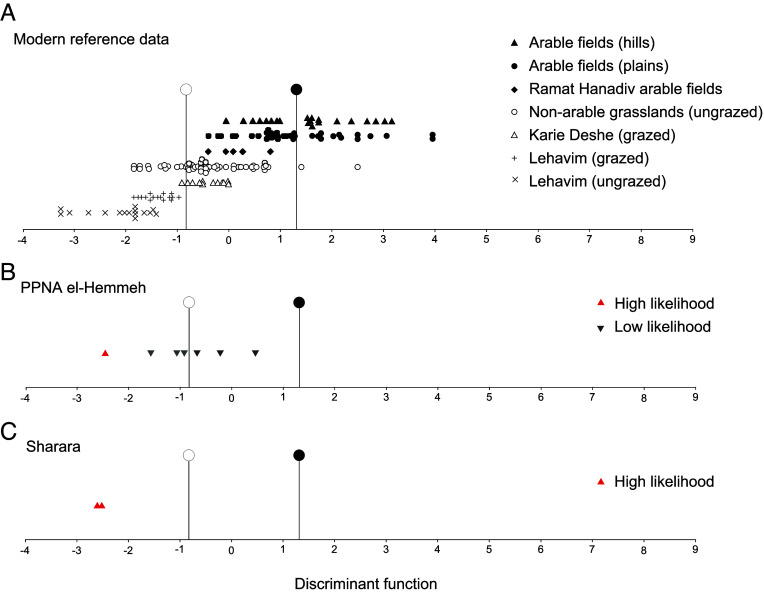
Discriminant analysis to distinguish between arable (tilled) and nonarable (untilled) habitats, with the larger closed and open symbols in each panel indicating the group centroids based on modern data. (*A*) Modern data from arable (closed symbols) and nonarable (open symbols) reported in Weide et al. (15). (*B*) Samples from PPNA el-Hemmeh. (*C*) Samples from PPNA Sharara. Archaeological samples coded according to likelihood that the sample represents crops and their associated weeds on the basis of crop processing analysis.

Particular traits of weed species have been shown to distinguish modern tilled arable fields and untilled wild cereal stands (15). These traits include duration of the flowering period, which predicts the ability of weed species to regerminate and recover from disturbance within a growing season, and (in perennials) root structure and regeneration from fragments (15). To test whether cultivation, specifically tillage, selected for larger grain size at our sites, we undertook functional weed ecological analysis to explore whether cereals were growing in tilled or untilled environments, using the model developed by Weide et al. (15) on the basis of flowering duration. Perennial weed remains were insufficient to include vegetative propagation in the model (*SI Appendix*, *Supporting Information Text*). This analysis situated archaeobotanical samples from PPNA Sharara (n = 2) and PPNA el-Hemmeh (n = 7) along an ecological continuum ranging from low to high soil disturbance (*SI Appendix*, *Supporting Information Text*). The results are presented in Fig. 5. Only samples containing cereal crops and associated weedy taxa in sufficiently high numbers were included in the analysis. No samples from LPPNB el-Hemmeh met the criteria for inclusion (*SI Appendix*, *Supporting Information Text*).

Our results demonstrate that low mechanical disturbance of soils featured at both PPNA Sharara and el-Hemmeh, indicating that cereals grew in soils that were not subject to systematic annual tillage (Fig. 5). A single PPNA sample from el-Hemmeh occurred within the range of higher mechanical soil disturbance associated with tillage, but the discriminant score for this sample (0.455) overlaps with modern-day nonarable (untilled) cereal habitats (Fig. 5*A*). Furthermore, based on crop processing analysis (*SI Appendix*, *Supporting Information Text*), this sample was considered to have a relatively low likelihood of representing crops and their associated weeds and probably reflects multiple sources of charred plant remains. We therefore infer that cereals at PPNA Sharara and el-Hemmeh grew in relatively low disturbance environments and were not subjected to systematic annual tillage.

## Discussion

Conventional models of plant domestication in southwest Asia call for scenarios where increased grain size, presumed to be a genetic adaptation to tillage, is selected for prior to loss of shattering, such that the presence of large, “domestic-sized” grains at PPNA sites provides evidence for cultivation (4, 6, 58). However, weed ecological analysis at PPNA Sharara and el-Hemmeh has demonstrated that cereals grew in conditions of low disturbance, comparable with those found in modern-day untilled wild cereal stands. We thus find no support for the hypothesis that the presence of larger “domestic-sized” grains at our PPNA sites reflects genetic selection for increased seed size under tillage. Instead, we interpret their occurrence as a plastic response to water availability, since stable carbon isotope analysis indicates a positive relationship between grain size and moisture. Our findings align with other studies that caution against attributing grain size variation solely to genetic selection under cultivation, emphasizing the diverse environmental and biological factors that influence this trait (2, 22, 58–61). These observations also underscore the limitations of using discrete grain size categories such as “wild-sized”, “intermediate-sized”, and “domestic-sized” to capture a dynamic evolutionary process.

While plasticity is important in determining grain size within a population, our results indicate that between populations its effect is mediated by genetic variation. This combination of factors explains why barley grains at Sharara are smaller on average than those at el-Hemmeh, despite growing in wetter conditions. In other words, there is a plastic and genetic component to grain size variation. The predominance of smaller barley grains at Sharara compared to el-Hemmeh plausibly reflects the existence of a local ecotype adapted to generally drier conditions, where even plants growing in relatively moist conditions were genetically predisposed toward small grain size. This evolutionary strategy has been documented in modern populations of wild barley in the region (32, 62).

Our data also highlight the need for an evidence-based redefinition of PDC, recognizing that PDC may have encompassed a diverse range of novel management practices other than systematic annual tillage. Plausible elements of a revised model of PDC include protection of cereal stands from herbivore grazing, periodic resowing (15, 63), and the harvesting of cereals growing under a spectrum of wet to dry conditions, as indicated by our findings. We do not exclude the possibility that variation in water availability at our PPNA sites reflects intentional human efforts to enhance productivity. However, when compared to modern barley cultivars, the Δ^13^C values recorded in our study generally indicate moderate to stressed water conditions for barley, ruling out significant levels of artificial irrigation (see Fig. 4 *A* and *B*, dashed lines for modern water status values). More broadly, and in comparison, to the Late Pleistocene, improved growing conditions may also have arisen in the Early Holocene through entirely nonanthropogenic means, such as increases in rainfall, groundwater levels, and atmospheric CO_2_ (64–66).

In contrast to the PPNA assemblages, our data from LPPNB el-Hemmeh are consistent with current archaeobotanical evidence for the establishment of domestic cereal populations across the Fertile Crescent by the LPPNB-Early Pottery Neolithic, with genetic selection for indehiscence evidenced by high proportions of nonshattering barley and emmer rachis. While it has not been possible to undertake weed ecological analysis at LPPNB el-Hemmeh, the predominance of nonshattering rachis provides indirect evidence for cultivation, as selection for this trait could only be maintained by sustained human intervention in the plants’ life cycle. However, the lack of a significant difference in barley grain size between PPNA and LPPNB el-Hemmeh underscores the inference that increased grain size was not selected for by cultivation per se.

We propose instead that genetic selection for increased grain size occurred after selection for nonshattering rachis. Cultivation and harvesting are known to relax selection pressures relating to dispersal, which tend to advantage small seeds that can disperse further, while simultaneously increasing selection pressures associated with yield optimization, including larger grain size (16, 67). Experimental studies have also demonstrated that larger-seeded genotypes might gain a selective advantage only when selection for dispersal is relaxed (68). We further hypothesize that this pattern represents an example of plasticity-led evolution in line with the EES. In this scenario, initial plastic increases in grain size in response to improved growing conditions in the PPNA could have generated the selective context in which human–cereal relationships intensified and nonshattering rachis evolved, ultimately leading to genetic selection for larger grain size. This interpretation is consistent with regional datasets of cereal grain measurements from southwest Asia, which show that grain size began to increase shortly before the appearance of nonshattering rachis, and continued beyond the point at which the nonshattering trait became fixed (69, 70).

Taken together, our findings indicate that the presence of larger “domestic-sized” grain at PPNA Sharara and el-Hemmeh is a function of developmental plasticity, linked, at least in part, to water availability. These results challenge the view that increased grain size necessarily signals genetic selection under cultivation, specifically tillage, and align with experimental evidence that has failed to demonstrate a relationship between burial depth and seed size (29, 30). Instead, we suggest a sequence in which plasticity-driven phenotypic changes resulting from improved growing conditions preceded and created the selective context for the genetic selection of nonshattering rachis, and subsequent selection for increased grain size. However, further work is needed to apply the integrated approach demonstrated here to other Early Holocene sites across the Levant and southwest Asia, enabling broader regional comparison. It is also necessary to clarify how plastic increases in grain size were translated into genetic changes over the course of cereal domestication, and how plasticity itself evolved during this process (71–73). By highlighting plasticity as an active evolutionary driver, our study underscores the need for continued investigation into the complex evolutionary dynamics involved in plant domestication, the constellation of traits this entailed and its wider ecological context (35, 39, 43, 72, 74–80), including the management practices involved in PDC.

## Materials and Methods

Archaeobotanical samples reported on in this study (n = 80) were analyzed at the School of Archaeology, University of Oxford. Field protocols for sampling and recovery are reported in the *SI Appendix*, *Supporting Information Text*. Charred macrobotanical remains were identified in comparison to modern reference material housed at the School of Archaeology, using a Leica MZ75 stereomicroscope at magnifications of 6× to 50× (*SI Appendix*, *Supporting Information Text*). Measurements of barley grains were obtained using a PixeLINK camera attached to a Nikon SMZ25 stereomicroscope and coupled to a PC, assisted by PixeLINK Capture and NIS-Elements D software, or by a Leica DFC495 camera attached to a Leica Z6 APO and coupled to a PC, assisted by LAS software.

A total of 143 cereal grains were individually analyzed for their carbon isotopic composition. This included 32 grains from Sharara, recovered primarily from Space 5 (48), 60 grains from PPNA el-Hemmeh, corresponding to seven distinct contexts and 51 grains from LPPNB el-Hemmeh corresponding to nine distinct contexts (*SI Appendix*, *Supporting Information Text*). Each grain was photographed in ventral, lateral, and cross-section views and assessed for their state after charring (81). Subsequently over 10% of the grains were analyzed using Fourier Transform Infrared Spectroscopy (FTIR) and their spectra assessed for potential contamination to decide on the appropriate pretreatment (82, 83). Each grain was then pretreated with 0.5M HCl, weighed into tin capsules, and analyzed on a Sercon EA-GSL at the University of Oxford’s Research Laboratory for Archaeology and the History of Art. Using international and in-house standards, the results were calibrated. The standard uncertainty at 1sd was ≤0.2‰ (mostly ≤0.1‰). Details of the *Methods and Materials* can be found in the *SI Appendix*, *Supporting Information Text*.

For weed ecological analysis, flowering duration was selected as the most useful trait for distinguishing between tilled and nontilled habitats following Weide et al. (15), All samples containing at least 30 cereal remains and 10 weed seeds identified to a taxonomic level sufficient to allow for the attribution of a functional-trait value were included in the analysis. Samples were coded according to how probable the crop–weed associations are based on crop processing analysis (*SI Appendix*, *Supporting Information Text*).

Flowering duration values were assigned to taxa using *Flora Palaestina* (*SI Appendix*, *Supporting Information Text* and Tables S4 and S5). For archaeobotanical taxa not identified to species, an average flowering duration value was calculated based on a list of possible species that are native to the region. Where flowering duration varied by more than two months among the possible species, we tested the minimum and maximum flowering durations in the analysis to see what effect this would have (*SI Appendix*, Fig. S8). The results of crop-processing analysis, conducted to determine how reliable samples were in terms of their crop–weed relationships, are reported in the *SI Appendix* (*SI Appendix*, Figs. S6 and S7 and Tables S2 and S3).

Note on data, materials, and software availability: Data processing conducted in MS Excel and R (84). Data analysis was undertaken in R Studio versions 4.1.2, 2023.12.1, and 2024.04.2+764. For weed ecological analysis, data were prepared in R studio with the discriminant analysis performed using IBM SPSS version 27. All archaeobotanical samples received a discriminant score, which was used to visualize the relative position of samples to each other by plotting the samples along the extracted discriminant function (in Microsoft Excel). Average attribute scores for each analyzed modern and archaeobotanical sample and the outputs of the DAs are provided in *SI Appendix*, Table S7. Final figures were prepared and formatted for clarity using Adobe Illustrator, without any modifications to the original data. The underlying data as CSV and XLSX files, an accompanying README file for each dataset, and an R Script to replicate all graphs and tables in the main text and *SI Appendix* are provided at the University of Oxford data repository (85, 86).

## Supplementary Material

Appendix 01 (PDF)

## Data Availability

Archaeobotanical data; stable isotope data; R scripts data have been deposited in Oxford University Research Archive [DOI: 10.5287/ora-bddyq9mj1 (86) and 10.5287/ora-n10ddbodq (85)].
